# Transabdominal Preperitoneal Repair for a Recurrent Inguinal Hernia After Kugel Hernioplasty Using the Pseudosac of Direct Inguinal Hernia: A Case Report

**DOI:** 10.7759/cureus.64610

**Published:** 2024-07-15

**Authors:** Takashi Deguchi, Goshi Fujimoto, Junya Shirai, Kentaro Saito

**Affiliations:** 1 Gastroenterological Surgery, Koga Community Hospital, Yaizu, JPN; 2 Gastroenterological Surgery, Koga Community Hospiral, Yaizu, JPN

**Keywords:** case report, pseudosac, transabdominal preperitoneal (tapp), kugel, recurrent inguinal hernia

## Abstract

Here, we report a case of laparoscopic trans-inguinal hernia repair (transabdominal preperitoneal repair or TAPP) for a recurrent inguinal hernia following direct Kugel surgery. A 71-year-old man underwent direct Kugel hernioplasty for a right inguinal hernia at another hospital 4 years prior to presentation. The patient subsequently underwent laparoscopic surgery using the TAPP technique, during which the abdominal cavity was visualized with a laparoscope, revealing a tubular mesh protruding towards the abdominal cavity with a direct and indirect hernia ring. Three months post-surgery, no recurrence was observed.

## Introduction

More than 20 million patients undergo groin hernia repair annually worldwide. Inguinal hernias are classified as direct and indirect hernias. Direct hernias occur due to weakness of the posterior wall of the inguinal canal, the tube through which the spermatic cord in men and the ligamentum teres uteri in women pass from the abdominal cavity to the subcutis. Indirect hernias occur within the inguinal canal from the internal inguinal ring to the external side of the inferior epigastric artery.

The Kugel technique, described by Kugel in 1999 [1], represents a repair method for adult inguinal hernias. It involves a direct approach to the dorsal inguinal canal, accessing the preperitoneal space without laparoscopic assistance. Here, the hernia ring is covered from the dorsal side using a specialized flat artificial mesh [1]. The postoperative quality of life of the patient is usually high [2], and surgeons typically feel comfortable once they become familiar with the technique. However, there have been several reports of recurrent cases [3-11], and a clear consensus on the optimal treatment of these recurrences has yet to emerge.

The European Hernia Society (EHS) recommends laparoscopic recurrent repair following previous open inguinal hernia surgery [12]. The laparoscopic posterior approach can be performed in the new layer even if the inguinal hernia recurs following the anterior approach, leading to a high rate of completion. So laparo-endoscopic recurrent inguinal hernia repair is recommended after failed anterior tissue repair, and laparoscopic surgery has a major advantage in treating recurrent inguinal hernias [13-15].

We present a case of transabdominal preperitoneal hernia repair (TAPP) in a patient with recurrent lesions following the direct application of Kugel's method.

## Case presentation

**Figure 1:**
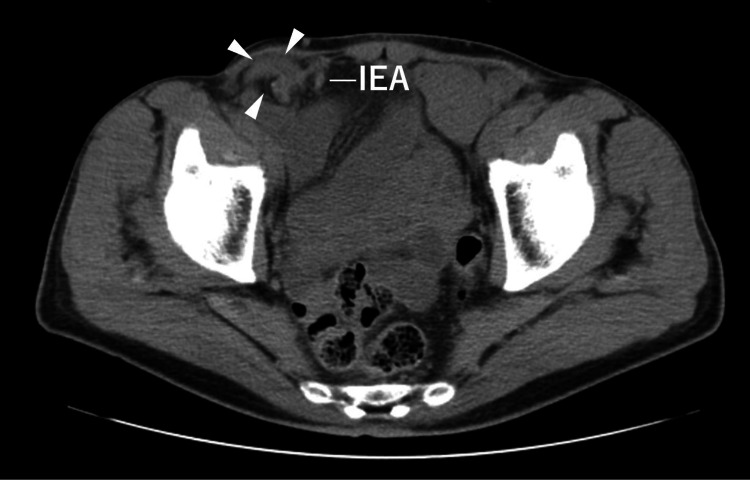
Preoperative plain abdominal computed tomography scan The small intestine (arrowheads) prolapses into the right inguinal hernia from outside the right inferior epigastric artery IEA, inferior epigastric artery

A 71-year-old man presented to our outpatient clinic with right inguinal distention persisting for 4 years and right inguinal pain for the past one week. The patient underwent direct Kugel hernioplasty for bilateral inguinal hernias at another hospital 4 years prior to presentation. He had a history of atrial fibrillation treated with catheter ablation. He had no history of smoking or alcohol consumption. Physical examination revealed a height of 170 cm, weight of 60 kg, and 5 cm transverse incisional skin wounds in both inguinal regions, with a fist-sized bulge noted in the right inguinal region. Plain computed tomography (CT) revealed an indirect inguinal hernia (Figure 1).

**Figure 2:**
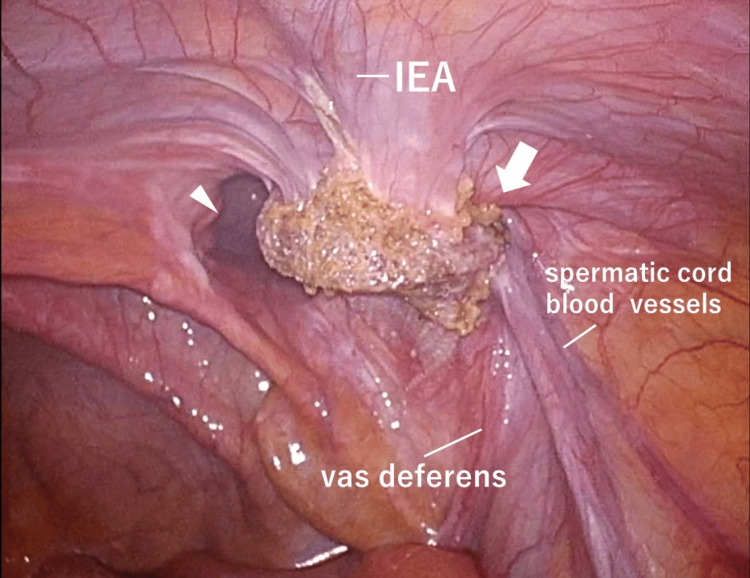
Laparoscopic findings The mesh was located medial to the hernia ring, and a direct (arrowhead) and indirect (arrow) hernia were revealed medial and lateral to the inferior epigastric artery, leading to the diagnosis of RL2M3. IEA, inferior epigastric artery

The patient subsequently underwent laparoscopic surgery using the TAPP technique for a recurrent right inguinal hernia following the direct Kugel's procedure. A 12-mm camera port was inserted through an umbilical incision using the open method, with 5-mm ports placed on the right and left sides of the abdomens. A 5-mm camera was used, and the patient was positioned in a supine head-down position with an insufflation pressure of 10 mm-Hg. Intra-operative findings revealed that the inlay mesh was inserted into the preperitoneal space from the medial side of the internal ring and was not sufficiently extended. Therefore, the mesh existed in a tubular shape involving the inferior epigastric artery, vas deferens, and spermatic cord blood vessels. A direct inguinal hernia was also identified within the mesh and diagnosed as RL2M3 (Figure 2) according to the EHS classification [2].

**Figure 3:**
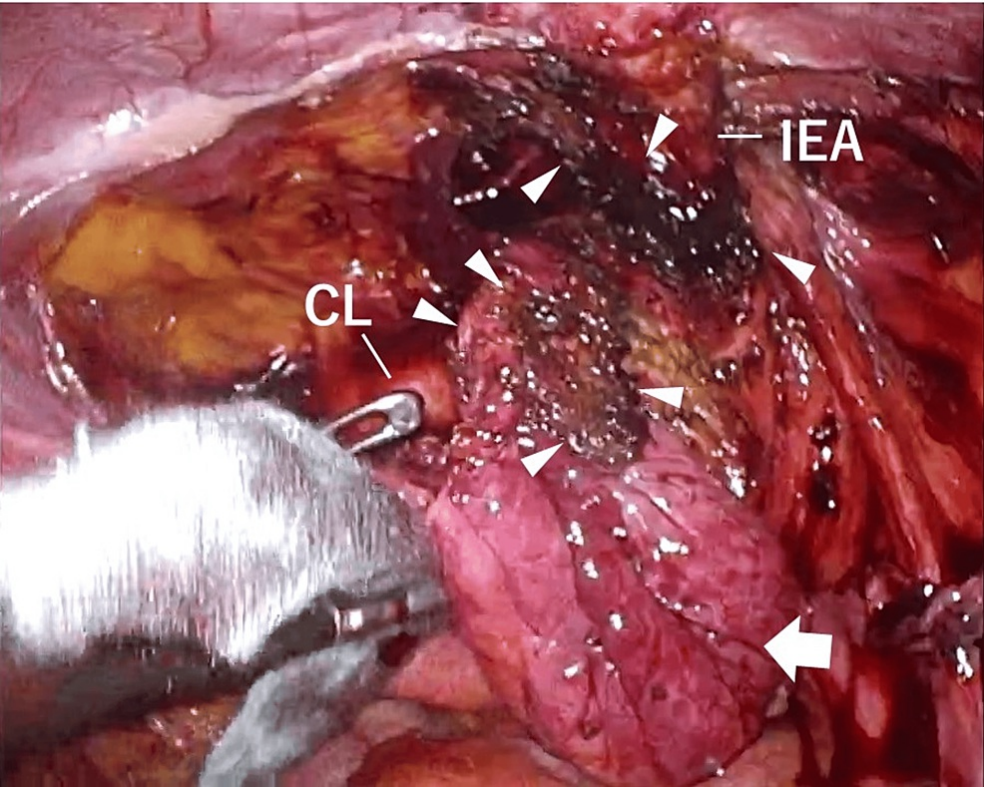
Detached preperitoneal cavity An incision was made across the Kugel mesh (arrowheads) to separate the peritoneum from the spermatic cord blood vessels and vas deferens along with the mesh, and pseudosac (arrow) was completely released by separating the preperitoneal area. IEA, inferior epigastric artery; CL, Cooper's ligament

The peritoneal membrane surrounding the mesh proved challenging to detach, and deploying the inlay mesh on the abdominal wall side was also difficult. Medial to the mesh, the preperitoneal cavity was dissected to create a pseudosac, revealing the Cooper's ligament and pubic tubercle (Figure 3).

**Figure 4:**
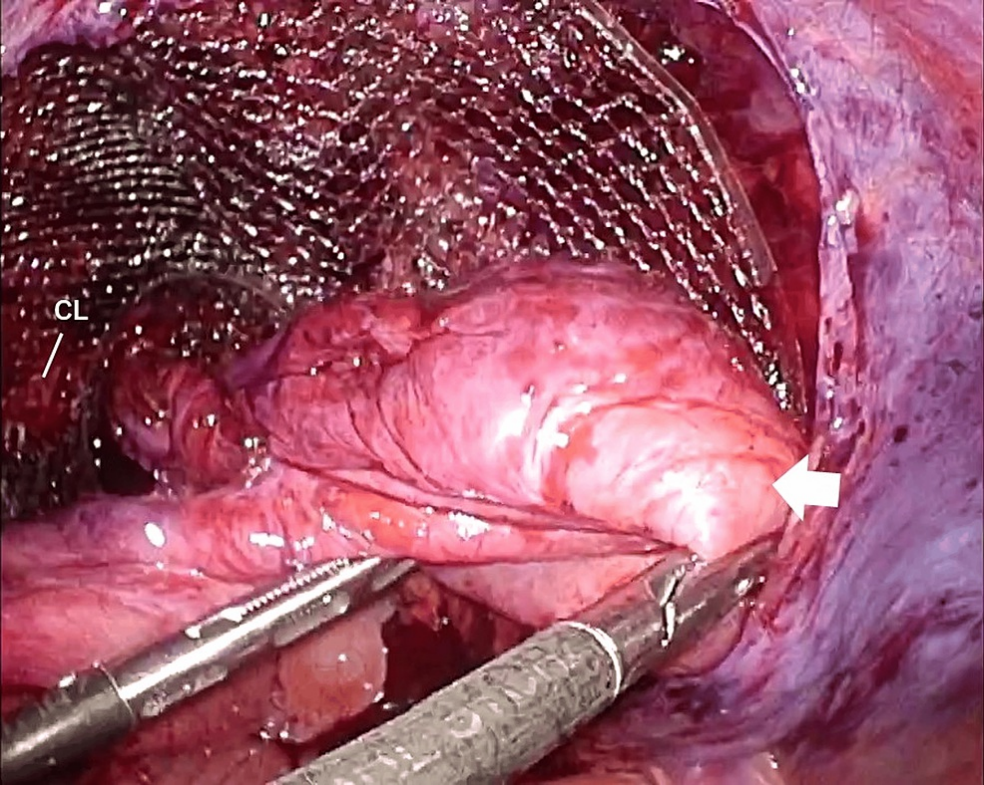
Lateral traction - the pseudosac (arrow) utilized for total peritoneal closure The pseudosac (arrow) was created by detaching the peritoneum of a direct hernia. CL, Cooper's ligament

Subsequently, an incision was made across the mesh to separate the peritoneum from the spermatic cord blood vessels and the vas deferens, along with the mesh. A space for mesh placement was prepared, and a mesh (3D MAX Light, size L, for the right, BARD, USA) was carefully positioned and secured in place and fixed using CapSure (BARD). Given the substantial size of the peritoneal defect, the prolapsed peritoneum, resembling a pseudosac of direct inguinal hernia, was gently pulled outward and reintegrated into the peritoneal defect (Figure 4).

**Figure 5:**
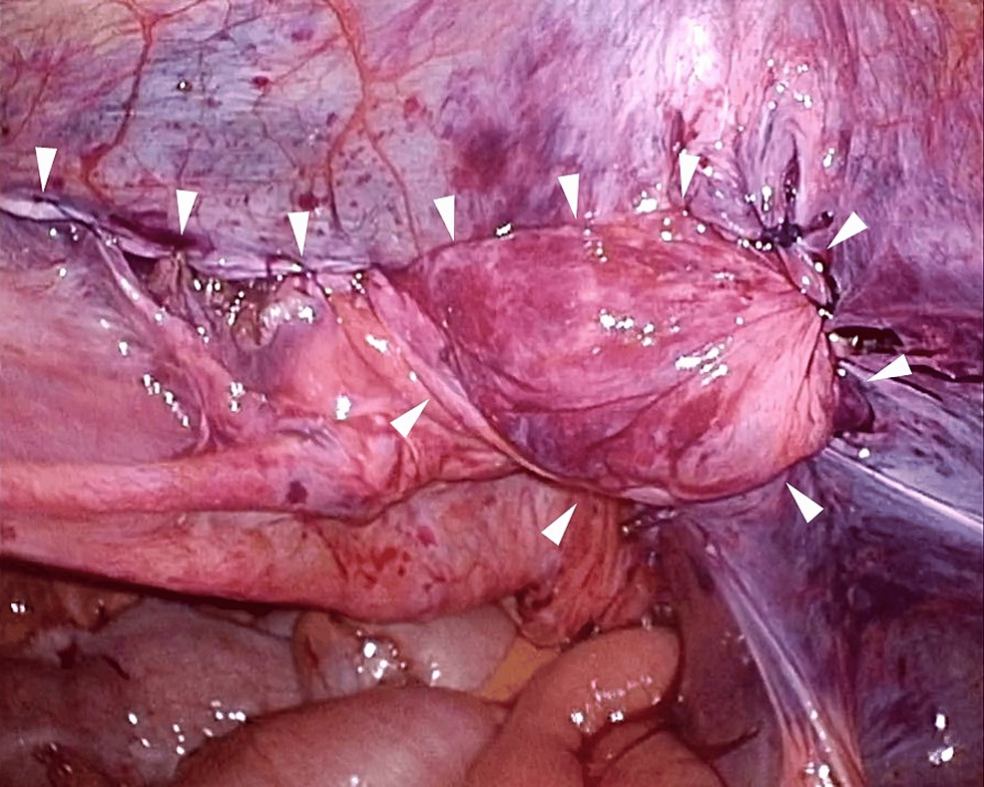
Total closure of the peritoneum Total closure of the peritoneum (arrowheads) was achieved by suturing the ventral and dorsal peritoneum to the pseudosac.

Subsequently, peritoneal closure was executed by suturing the detached ventral and dorsal peritoneum to the integrated peritoneum (Figure 5).

The total duration of the operative procedure was 4 h, with an insufflation time of 3 h 48 min, and blood loss was 20 ml. At the 3-month follow-up visit, the patient reported no complications or recurrences.

Written informed consent was obtained from the patient for the publication of this case report and the accompanying images.

## Discussion

Preperitoneal repair by inguinal incision was first reported by Usher et al. in 1959 [16] and is well described by Rives [17] and Wantz [18]. However, flat mesh placement in the anterior peritoneal cavity has not been widely used because it requires several fixed sutures, making the surgical technique complicated. This issue was addressed by Kugel in 1999 with preperitoneal repair using a mesh featuring a shape memory ring [1]. Subsequently, transinguinal preperitoneal repair using the anterior approach, which is more familiar to general surgeons, was initiated.

The recurrence rate after using Kugel's method has been reported to be low, ranging from 0% to 7.7% [3-11]. Recurrence patterns include indirect inguinal recurrence, which occurs when the hernia ring is not covered because the mesh is displaced medially without deformation, and direct inguinal recurrence, which happens when the mesh is not adequately spread. In this case, the mesh was retained in the preperitoneal space without spreading adequately, resulting in both direct and indirect inguinal recurrences. Although bilateral recurrent hernias are rare, and, to the best of our knowledge, no such cases have been reported.

According to the EHS guidelines [12], we performed TAPP to repair recurrence after anterior repair. TAPP repair of post-Kugel recurrences requires extensive peritoneal defects for dissection of the preperitoneal space due to strong adhesions between the mesh and the peritoneum. Alternative methods of peritoneal closure may be necessary due to the strain on the peritoneum, and achieving total closure may not always be possible. Therefore, there are reports of using the median umbilical folds [3] and achieving peritoneal closure using an anti-adhesion-coated mesh. Although we successfully achieved total closure of the peritoneum using a pseudosac for direct hernia, it is important to consider the use of an anti-adhesion-coated mesh for the repair procedure, or to convert the Lichtenstein technique.

## Conclusions

In conclusion, TAPP is not only efficacious but also indispensable in addressing recurrent hernias subsequent to the Kugel procedure. The intricate nature of mesh adhesions and the anticipated extensive peritoneal defects underscore the imperative prosthetic materials to enable preperitoneal closure, such as the extra peritoneum of the hernial sac or median umbilical folds.

Moreover, the successful resolution of this case emphasizes the importance of meticulous surgical technique and highlights the significance of further exploration into innovative approaches to optimize outcomes in recurrent inguinal hernia repair.

## References

[1] Kugel RD (1999). Minimally invasive, nonlaparoscopic, preperitoneal, and sutureless, inguinal herniorrhaphy. Am J Surg.

[2] Ceriani V, Faleschini E, Bignami P, Lodi T, Roncaglia O, Osio C, Sarli D (2005). Kugel hernia repair: open "mini-invasive" technique. Personal experience on 620 patients. Hernia.

[3] Wang C, Lin R, Lin X, Lu F, Chen Y, Huang H (2022). Transabdominal preperitoneal repair for a recurrent inguinal hernia after Kugel procedure using the medial umbilical ligament: a case report. J Minim Access Surg.

[4] Takayama S, Sakamoto M, Takeyama H (2010). Laparoscopic inguinal hernia repair with the Composix Kugel Patch. Int Surg.

[5] Lin R, Lin X, Lu F (2018). A 12-year experience of using the Kugel procedure for adult inguinal hernias via the internal ring approach. Hernia.

[6] Chiang HC, Chen PH, Chen YL (2015). Inguinal hernia repair outcomes that utilized the modified Kugel patch without the optional onlay patch: a case series of 163 consecutive patients. Hernia.

[7] Van Nieuwenhove Y, Vansteenkiste F, Vierendeels T, Coenye K (2007). Open, preperitoneal hernia repair with the Kugel patch: a prospective, multicentre study of 450 repairs. Hernia.

[8] Li J, Zhang Y, Hu H, Tang W (2008). Preperitoneal groin hernia repair with Kugel patch through an anterior approach. ANZ J Surg.

[9] Zhou X (2013). Comparison of the posterior approach and anterior approach for a Kugel repair of treatment of inguinal hernias. Surg Today.

[10] Yaguchi Y, Inaba T, Kumata Y, Horikawa M, Kiyokawa T, Fukushima R (2018). Two cases of early recurrence after transabdominal preperitoneal inguinal hernia repair. Asian J Endosc Surg.

[11] Schroder DM, Lloyd LR, Boccaccio JE, Wesen CA (2004). Inguinal hernia recurrence following preperitoneal Kugel patch repair. Am Surg.

[12] (2018). International guidelines for groin hernia management. Hernia.

[13] Li J, Ji Z, Li Y (2014). Comparison of laparoscopic versus open procedure in the treatment of recurrent inguinal hernia: a meta-analysis of the results. Am J Surg.

[14] Köckerling F, Bittner R, Kuthe A (2017). Laparo-endoscopic versus open recurrent inguinal hernia repair: should we follow the guidelines?. Surg Endosc.

[15] Yang J, Tong da N, Yao J, Chen W (2013). Laparoscopic or Lichtenstein repair for recurrent inguinal hernia: a meta-analysis of randomized controlled trials. ANZ J Surg.

[16] Usher FC, Fries JG, Ochsner JL, Tuttle LL (1959). Marlex mesh, a new plastic mesh for replacing tissue defects. II. Clinical studies. AMA Arch Surg.

[17] Rives J (1967). Surgical treatment of the inguinal hernia with dacron patch. Int Surg.

[18] Wantz GE (1993). The technique of giant prosthetic reinforcement of the visceral sac performed through an anterior groin incision. Surg Gynecol Obstet.

